# Crosstalk between Bone and Nerves within Bone

**DOI:** 10.1002/advs.202003390

**Published:** 2021-02-10

**Authors:** Qian‐Qian Wan, Wen‐Pin Qin, Yu‐Xuan Ma, Min‐Juan Shen, Jing Li, Zi‐Bin Zhang, Ji‐Hua Chen, Franklin R. Tay, Li‐Na Niu, Kai Jiao

**Affiliations:** ^1^ State Key Laboratory of Military Stomatology and National Clinical Research Center for Oral Diseases and Shaanxi Key Laboratory of Stomatology Department of Prosthodontics School of Stomatology The Fourth Military Medical University Xi'an Shaanxi 710032 China; ^2^ College of Graduate Studies Augusta University Augusta GA 30912 USA

**Keywords:** bioactive factors, bone metabolism, crosstalk, nerve growth, peripheral nerves

## Abstract

For the past two decades, the function of intrabony nerves on bone has been a subject of intense research, while the function of bone on intrabony nerves is still hidden in the corner. In the present review, the possible crosstalk between bone and intrabony peripheral nerves will be comprehensively analyzed. Peripheral nerves participate in bone development and repair via a host of signals generated through the secretion of neurotransmitters, neuropeptides, axon guidance factors and neurotrophins, with additional contribution from nerve‐resident cells. In return, bone contributes to this microenvironmental rendezvous by housing the nerves within its internal milieu to provide mechanical support and a protective shelf. A large ensemble of chemical, mechanical, and electrical cues works in harmony with bone marrow stromal cells in the regulation of intrabony nerves. The crosstalk between bone and nerves is not limited to the physiological state, but also involved in various bone diseases including osteoporosis, osteoarthritis, heterotopic ossification, psychological stress‐related bone abnormalities, and bone related tumors. This crosstalk may be harnessed in the design of tissue engineering scaffolds for repair of bone defects or be targeted for treatment of diseases related to bone and peripheral nerves.

## Graphical abstract

Some clues are given during biological processes as well as pathological disorders to allow the detection of the communication between bone and nerves within bone. The pattern of strings on the hand created by nature are intriguing and waiting for the wise play.

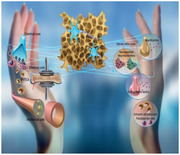

## 1 Introduction

Studies on the intersection of bone mass, body weight, and gonadal function in the early 2000s provided insight on how the neural system regulates bone remodeling. Augmentation of vertebral trabecular bone mass was identified in obese mice and hypogonadic ones that were deficient in leptin or its receptors, an adipose tissue‐derived hormone that regulates food intake and energy expenditure. In vivo observations suggest that leptin acts through the central nervous system to mediate bone metabolism.^[^ ^1^ ^]^ Changes in the central nervous system can induce alterations in bone mass through humoral mechanisms such as mediation of plasma calcium, as well as thyrotropic, hypothalamo‐pituitary‐corticotropic, somatotropic or gonadotropic output.^[^ ^2^ ^]^ Humoral mechanism that is initiated and orchestrated by central nervous system has been perceived as a fundamental regulatory pathway from nervous system to bone metabolism.

Coincidentally, another regulatory pathway of bone metabolism also stems from leptin. In 2002, leptin was found to modulate bone mass by altering the sympathetic tone after acting on the hypothalamus. This causes peripheral nerves to release norepinephrine into the local microenvironment, which in turn activates *β*‐adrenergic receptors expressed by osteoblasts.^[^ ^3^, ^4^ ^]^ Other molecules such as neuromedin U also regulate bone mass through signals processed in the hypothalamus. Those signals, in turn, modulate sympathetic signals conveyed through peripheral nerves.^[^ ^5^ ^]^ With more attention paid to the local microenvironment within bone, the regulatory role of the peripheral neural system on bone metabolism slowly comes to light. Regulation of bone reshaping via peripheral nerves is now perceived as another pathway of nerve regulation on bone metabolism.

Intrabony nerves are well distributed in skeleton, including cortical and trabecular bone, bone marrow and periosteum.^[^ ^6^, ^7^, ^8^ ^]^ Peripheral nerves within the skeleton are sensory nerves and motor nerves. Intrabony motor nerves are mainly visceral motor nerves, which are further divided into adrenergic and cholinergic nerves according to their released neurotransmitters. These peripheral nerves communicate with the skeleton to regulate bone metabolism through nerve‐resident cells, locally‐released neurotransmitters, neuropeptides, axon guidance factors and neurotrophins. Peripheral regulation, the second regulatory pathway of bone metabolism by the nervous system, will be focused in the present review.

**Figure 1:**
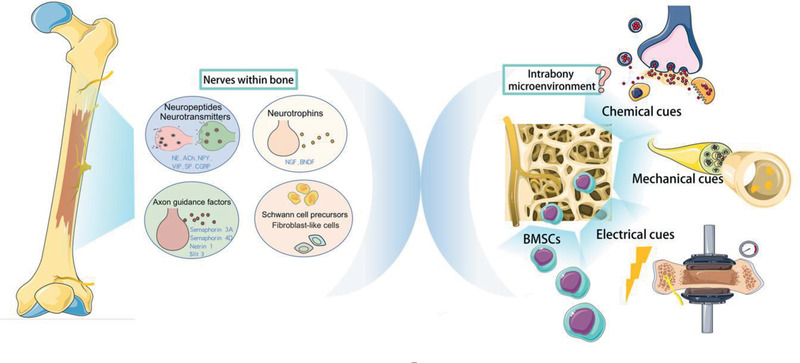
Crosstalk between bone and peripheral nerves within the skeleton. NE: norepinephrine; ACh: acetylcholine; NPY: neuropeptide Y; VIP: vasoactive intestinal peptide; SP: substance P; CGRP: calcitonin gene‐related peptide; BMSCs: bone marrow derived stroma cells. [Adopted from SMART Servier Medical ART under the terms of the CC‐BY Creative Commons Attribution 3.0 Unported license. (http://creativecommons.org/licenses/by/3.0/).]

The skeleton, a multifunctional complex, also exerts regulatory control of the peripheral nerves within bone. Bone harbors peripheral nerves at the physical level, provides bioactive factors and electrical cues into intrabony microenvironment, and also offers related cells participating nerve repair. However, this aspect has long been overlooked in prior literatures. A large ensemble of chemical, mechanical and electrical cues provided by the skeleton to orchestrate the physiological activities of intrabony nerves, as well as bone marrow stromal cells within skeletal microenvironment potentially serving as an extra cell source for nerve repair, will be summarized in this review (**Figure** **1**).

Crosstalk between bone and nerves is also involved in bone diseases such as osteoporosis, osteoarthritis, heterotopic ossification, psychological stress‐related bone abnormalities and bone tumors. The benefits of this crosstalk may be harnessed for developing novel scaffolds for bone repair. In the present review, communication between bone and peripheral nerves will be comprehensively analyzed, supported by in vivo evidence derived from physiological and pathological conditions.

## 2 Possible Signals from Intrabony Nerves to Bone

Bone metabolism is regulated by signals generated by intrabony nerves. These signals mediate bone mass and maintain the micro‐ and macro‐architecture of bone through regulating bone deposition by osteoblasts and resorption by osteoclasts.^[^ ^9^, ^10^ ^]^ Nerves within bone perform an important role as a conductor, sometimes expediting osteogenesis while sometimes impeding the process to keep bone metabolism in harmony. Such a process is vigilantly coordinated by peripheral nerves in a spatiotemporal‐orchestrated manner, during physiologic remodeling as well as repair of bone injury.^[^ ^11^ ^]^ Nerve fibers are frequently identified in the trabecular bone, periosteum and the callus that forms around the ends of broken bone after bone fracture.^[^ ^12^ ^]^ Nerves regulate bone metabolism via secretion of neurotransmitters, neuropeptides, neurotrophins, neuronal guidance factors, and participation of nerve‐resident cell components (e.g., Schwann cell precursors). The preponderance of these biological factors within the microenvironment, together with the expression of their receptors in cells of different lineages, are evidence of bilateral homeostasis between bone and nerves. Neurotransmitters, neuropeptides, neurotrophins, and neuronal guidance factors, which are regulated by skeletal tissues as well as neural peripheral nerves (**Table** **1**), attach to receptors expressed by cells of the nervous system as well as bone lineage cells (**Table** **2**). These signaling molecules function as mediators between the nervous system and the skeleton.

**Table 1:** Roles of bone and peripheral nerves in regulating biological factors within the bone microenvironment. NE: norepinephrine. MAO: monoamine oxidase. ACh: acetylcholine. VAChT: vesicular ACh transporter. AChE: acetylcholine esterase. BChE: butyrylcholinesterases. CarAT: carnitine acetyl transferase. CGRP: calcitonin gene‐related peptide. VIP: vasoactive intestinal peptide. NPY: neuropeptide Y. SP: substance P. NGF: nerve growth factor. BDGF: Brain‐derived neurotrophic factor

Bioactive factors		Roles of peripheral nervous system	Roles of skeleton
Neurotransmitters and neuropeptides	NE	Synthesis and secretion NE: secreted by sympathetic adrenergic nerves^[^ ^17^ ^]^	Regulation 1) enzymes MAO*α* and MAO*β*: expressed in osteoblast precursor cells and in fully differentiated osteoblasts^[^ ^25^, ^31^ ^]^ 2) NE transporter: expressed in differentiated osteoblasts^[^ ^25^ ^]^
	ACh	Synthesis and secretion ACh: secreted by cholinergic nerves^[^ ^46^ ^]^	Regulation 1) VAChT, the choline transporter, and CarAT: expressed in osteoblasts^[^ ^48^, ^49^ ^]^ 2) AChE and BChE: expressed in osteoblasts^[^ ^48^, ^49^ ^]^
	CGRP	Synthesis and secretion CGRP: secreted by sensory nerves^[^ ^61^, ^62^ ^]^	Regulation CGRP: expressed in human osteosarcoma cells and primary human osteoblasts^[^ ^200^ ^]^
	VIP	Synthesis and secretion VIP: secreted by cholinergic nerves^[^ ^80^, ^81^ ^]^	Regulation VIP: expressed in isolated pure populations of osteoclasts^[^ ^199^ ^]^
	NPY	Synthesis and secretion NPY: secreted by sympathetic adrenergic nerves^[^ ^76^ ^]^	Regulation NPY: produced by osteocytes and osteoblasts^[^ ^202^ ^]^
	SP	Synthesis and secretion SP: secreted by sensory nerves^[^ ^69^, ^70^ ^]^	Regulation SP and the receptor NK‐1: expressed in osteoblasts and osteocytes especially under mechanical stimulation during exercise^[^ ^201^ ^]^
Axon guidance factors	Sema3A	Synthesis and secretion Sema3A: secreted by a specific subset of nerves including sensory nerves^[^ ^84^, ^89^ ^]^	Synthesis and secretion Sema3A: secreted by osteoblast lineage cells and expressed in bone cell lineages including chondrocytes, osteoblasts, and osteoclasts^[^ ^86^, ^137^ ^]^
	Sema4D	Synthesis and secretion Sema4D and its receptors plexin‐B1, plexin‐B2: expressed in embryonic dorsal root ganglion^[^ ^91^ ^]^	Synthesis and secretion Sema4D: strongly expressed in osteoclasts, with no evidence of its expression in osteoblasts^[^ ^87^, ^94^, ^96^, ^144^ ^]^
	Netrin‐1	Synthesis and secretion Netrin‐1: continuously expressed in the nervous system. Expression in Schwann cells up‐regulated during nerve repair^[^ ^99^ ^]^	Synthesis and secretion Netrin‐1: produced by osteoblasts and osteoclast. Expression of Netrin‐1 in osteoblasts was found to be 200‐fold higher than that in osteoclasts^[^ ^100^, ^103^, ^157^ ^]^
	Slit‐3	Synthesis and secretion Slit‐3: expressed in the cell bodies and axons of both motor and sensory neurons, satellite cells of the dorsal root ganglion, Schwann cells and fibroblasts of peripheral nerves^[^ ^106^ ^]^	Synthesis and secretion Slit‐3: secreted by osteoclasts and osteoblasts. Production increasing during osteoclast differentiation^[^ ^109^, ^110^ ^]^
Neurotrophins	NGF	Synthesis and secretion NGF: highly concentrated in the nervous system during nerve development or regeneration. Secreted by Schwann cells of peripheral nerves^[^ ^113^ ^]^	Synthesis and secretion NGF: expressed in bone marrow stromal cells, osteoblasts as well as osteoblastic cell lines. Expression of NGF is upregulated during proliferation or upon loading^[^ ^122^, ^167^, ^168^, ^169^ ^]^
	BDNF	Synthesis and secretion BDNF: highly concentrated in the nervous system during nerve development or regeneration. Secreted by Schwann cells of peripheral nerves^[^ ^113^ ^]^	Synthesis and secretion BDNF and its receptor TrkB: expressed in fracture bone tissues during early bone formation. Concentrated in endothelial and osteoblastic cells^[^ ^122^, ^170^, ^171^ ^]^

**Table 2:** Expression of receptors of biological factors in bone lineage cells and cells in nervous system. NE: norepinephrine. ACh: acetylcholine. NGF: nerve growth factor. BDGF: Brain‐derived neurotrophic factor

Corresponding molecule	Receptor	Bone cell lineage	Nerve cell lineage
NE	*α*1AR	Immature osteoblasts: • Rat bone marrow mesenchymal cells^[^ ^20^ ^]^ • Mouse sarcoma C3H10T1/2 cells^[^ ^21^ ^]^ • Mouse MC3T3 cells^[^ ^22^ ^]^ • Human fetal long bone‐derived osteoblasts^[^ ^23^ ^]^ • Mouse bone marrow stromal cells^[^ ^21^ ^]^ Differentiated osteoblasts: • Mouse bone marrow stromal cells^[^ ^21^ ^]^	• Rat cutaneous nerve fibers that survive partial ligation or chronic constriction of the sciatic nerve^[^ ^185^, ^186^ ^]^ • Human cutaneous nerve fibers with complex regional pain syndrome^[^ ^187^ ^]^
	*α*2AR	Chondrocytes: • Mouse growth plate chondrocytes (IHC)^[^ ^24^ ^]^ Immature osteoblasts: • Mouse MC3T3 cells^[^ ^24^ ^]^	• Mouse sympathetic neurons^[^ ^188^ ^]^ • Cell bodies and axons of rat mesoprefrontal dopaminergic neurons^[^ ^189^ ^]^
	*β*1AR	Immature osteoblasts: • Human osteosarcoma SaOS2 cells^[^ ^33^ ^]^ • Human osteosarcoma TE‐85 cells^[^ ^33^ ^]^ • Human osteosarcoma OSH‐4 cells^[^ ^33^ ^]^	• Mouse induced pluripotent stem undergoing neural differentiation^[^ ^190^ ^]^ • Mouse sympathetic‐ and motor‐neurons^[^ ^188^ ^]^ • Mouse GABAergic interneurons in the medial prefrontal cortex, including parvalbumin (PV)‐, calretinin (CR)‐, calbindin D‐28k (CB)‐, somatostatin (SST)‐ and Reelin‐immunoreactive (ir) interneurons^[^ ^191^ ^]^
	*β*2AR	Osteoclasts: • Differentiated bone marrow macrophages and Raw 264.7 cells^[^ ^33^ ^]^ Immature osteoblasts: • Mouse MC3T3 cells^[^ ^24^ ^]^ • Human fetal long bone‐derived osteoblasts^[^ ^24^ ^]^ • Human osteosarcoma MG63 cells^[^ ^33^ ^]^ • Human osteosarcoma TE‐85 cells^[^ ^33^ ^]^ • Rat ROS 17/2.8 cells^[^ ^34^ ^]^ • Human periosteum‐derived osteoblastic SaM‐1 cells^[^ ^35^ ^]^ • Human osteosarcoma HOS cells^[^ ^35^ ^]^ • Mouse calvarial osteoblasts^[^ ^34^ ^]^ Osteocytes: • Mouse IHC^[^ ^34^ ^]^	• Mouse GABAergic interneurons in the medial prefrontal cortex, including parvalbumin (PV)‐, calretinin (CR)‐, calbindin D‐28k (CB)‐, somatostatin (SST)‐ and Reelin‐immunoreactive (ir) interneurons^[^ ^191^ ^]^
	*β*3AR	Immature osteoblasts: • Human primary osteoblasts^[^ ^35^ ^]^ • Mouse mesenchymal stem cells undergoing osteogenic differentiation^[^ ^36^ ^]^	• Rat small‐diameter tyrosine hydroxylase and vesicular mono‐amine transporter immuno‐reactive (THIR and vmat‐IR) neurons^[^ ^192^ ^]^ • Human acetylcholine‐containing nerve fibers of the urinary bladder^[^ ^193^ ^]^
ACh	nAChR	Monocytes: • Mouse bone marrow‐derived monocytes^[^ ^47^ ^]^ Osteoclasts: • Differentiated mouse bone marrow‐derived osteoclasts^[^ ^47^ ^]^ • Differentiated RAW264.7 cells^[^ ^48^ ^]^ Immature osteoblasts: • Mouse calvarial osteoblasts^[^ ^47^ ^]^ • Human primary osteoblasts and MG63 osteosarcoma cells^[^ ^49^ ^]^ • Human osteosarcoma SaOS2 cells^[^ ^50^ ^]^ • Mouse MC3T3 cells^[^ ^50^, ^51^ ^]^ Differentiated osteoblasts: • Mouse MC3T3 cells^[^ ^50^, ^51^ ^]^ • Mouse calvarial osteoblasts^[^ ^51^, ^52^ ^]^	• Rat sympathetic neurons of superior cervical sympathetic ganglion^[^ ^194^ ^]^ • Rat prepositus hypoglossi nuclei neurons^[^ ^195^ ^]^ • Rat dorsal root ganglia^[^ ^196^ ^]^
	mAChR	Monocytes: • Mouse bone marrow‐derived monocytes^[^ ^47^ ^]^ Osteoclasts: • Differentiated mouse bone marrow‐derived osteoclasts^[^ ^47^ ^]^ Immature osteoblasts: • Mouse calvarial osteoblasts^[^ ^47^ ^]^ • Human osteosarcoma SaOS2 cells^[^ ^50^ ^]^ • Mouse MC3T3 cells^[^ ^50^, ^51^ ^]^ Differentiated osteoblasts: • Mouse MC3T3 cells^[^ ^50^, ^51^ ^]^ • Mouse calvarial osteoblasts^[^ ^51^, ^52^ ^]^	• Pigeon vestibular nerve fiber terminals (myelin sheaths and Schwann cells)^[^ ^197^ ^]^ • Pigeon vestibular (Scarpa's) ganglion^[^ ^197^ ^]^
Sema3A	Neuropilin‐1 & Plexin‐A	Osteoclasts: • Human polyethylene particle‐stimulated peripheral blood mononuclear cell‐derived osteoclasts^[^ ^85^ ^]^ • Human peripheral blood mononuclear cell‐derived osteoclasts^[^ ^86^ ^]^ • Mouse bone marrow cell‐derived primary osteoclast^[^ ^87^ ^]^ Immature osteoblasts: • Mouse calvarial bone‐derived osteoblasts^[^ ^86^ ^]^ • Mouse bone marrow cell‐derived osteoblast precursors^[^ ^87^ ^]^ • Murine preosteoblast MC3T3‐E1 subclone 14 cells^[^ ^88^ ^]^	• Mouse callosal axons derived from the cingulate and neocortex^[^ ^139^ ^]^ • The proximal stump of mouse trigeminal ganglion neurons after transection of the inferior alveolar nerve^[^ ^140^ ^]^ • Rat dorsal root ganglia after a unilateral dorsal rhizotomy or sciatic nerve transection^[^ ^141^ ^]^ • Zebrafish spinal motor neurons^[^ ^142^ ^]^ • Mouse cranial crest cells^[^ ^143^ ^]^
Sema4D	Plexin‐B1 Plexin‐B2	Osteoclasts: • Mouse bone marrow cell‐derived primary osteoclast^[^ ^87^ ^]^ Immature osteoblasts: • Mouse bone marrow cell‐derived osteoblast precursors^[^ ^87^ ^]^ • Mouse primary calvarial osteoblasts^[^ ^93^ ^]^ • Mouse MC3T3‐E1 cells^[^ ^93^ ^]^	• Mouse or rat neurons and glia in the developing hippocampus^[^ ^145^ ^]^ • Mouse neural progenitors in the developing cortex^[^ ^146^ ^]^ • Certain cells in rat sciatic nerve after crush injury^[^ ^147^ ^]^
Netrin‐1	Unc5b	Osteoclasts: • Mouse monocyte macrophage‐derived osteoclast^[^ ^100^ ^]^ • Raw 264.7 cells^[^ ^101^ ^]^ • Human bone marrow myeloid precursor derived osteoclasts^[^ ^101^ ^]^ Immature osteoblasts: • Human adipose‐derived stem cells undergoing osteogenic differentiation^[^ ^102^ ^]^ • Mouse pre‐osteoblastic cell line MC3T3‐E1 cells^[^ ^103^ ^]^	• Mouse median nerve distal to the transection site after transection and microsurgical repair^[^ ^155^ ^]^ • RSC96 Schwann cells (an immortalized rat Schwann cell line^[^ ^156^ ^]^
Slit‐3	ROBO1	Osteoclasts: • Mouse bone marrow macrophage‐derived osteoclast precursors^[^ ^107^ ^]^ Immature osteoblasts: • Human fibroblast‐like synovial cells in rheumatoid arthritis undergoing osteogenic differentiation^[^ ^108^ ^]^	• Mouse commissural neurons in the vertebrate spinal cord^[^ ^148^ ^]^ • Mouse cranial neural crest cells^[^ ^151^ ^]^ • Mouse Schwann cells^[^ ^150^ ^]^ • Certain cells in mouse sciatic nerves during regeneration^[^ ^151^ ^]^ • Mouse dorsal root ganglion neurons^[^ ^152^ ^]^
NGF	TrkA	Chondrocytes: • Human cultured chondrocytes harvested from articular cartilages of knee joints in healthy and OA patients^[^ ^115^ ^]^ • Mouse differentiating chondrocytes in the central core of the limb bud and in the epiphyseal growth plate of the bone^[^ ^116^ ^]^ Immature osteoblasts: • Canine osteosarcoma cells^[^ ^117^ ^]^ Osteoclasts: • Rat osteoclasts in the periodontal ligament^[^ ^118^ ^]^	• Mouse hippocampal neurons^[^ ^159^ ^]^ • Mouse NeuN^+^ cells, GAP‐43^+^ axons, GFAP^+^ cells, Arginase1^+^ cells, and Mac3^+^ cells in the inflammatory lesions in the spinal cord^[^ ^160^ ^]^ • Human interspinal schwannoma cells^[^ ^161^ ^]^ • Adult rat adrenal medullary pheochromocytoma PC12 cells^[^ ^162^ ^]^ • Mouse dorsal root ganglion neurons^[^ ^162^ ^]^ • Mouse sensory neurons (subpopulations of dorsal root ganglion neurons)^[^ ^163^ ^]^ • Certain cells in mouse nerve fibers located nearby NGF^+^ blood vessels^[^ ^119^ ^]^ • Mouse skeletal sensory nerves^[^ ^164^ ^]^
	p75NTR	Chondrocytes: • Mouse chondrocytes located in the deep and middle zone of the articular cartilage^[^ ^119^ ^]^ Immature osteoblasts: • Murine multipotent C3H10T1/2 mesenchymal stem cells^[^ ^120^ ^]^ • Mouse marrow stromal cells undergoing osteoblastic differentiation^[^ ^121^ ^]^	• Human interspinal schwannoma cells^[^ ^161^ ^]^ • Mouse sensory neurons (subpopulations of dorsal root ganglion neurons)^[^ ^163^ ^]^ • Certain cells in mouse nerve fibers located nearby NGF^+^ blood vessels^[^ ^119^ ^]^
BDNF	TrkB	Chondrocytes: • Mouse differentiating chondrocytes in the central core of the limb bud and in the epiphyseal growth plate of the bone^[^ ^116^ ^]^ Immature osteoblasts: • Mouse osteoblastic MC3T3‐E1 cells^[^ ^122^ ^]^ Osteoclasts: • Rat osteoclasts in the periodontal ligament^[^ ^118^ ^]^ Cementoblasts: • Human cementoblast‐like cells^[^ ^129^ ^]^	• Certain cells in the frontal cortex, hippocampus, cerebellar cortex, pituitary gland, visual system, and hypothalamus^[^ ^173^, ^174^ ^]^ • Certain cells among injured rat spinal cord tissue^[^ ^175^ ^]^ • Mouse sensory neurons (subpopulations of dorsal root ganglion neurons)^[^ ^163^ ^]^

### 2.1 Neurotransmitters and Neuropeptides

Synapses have not been identified within bone.^[^ ^13^, ^14^ ^]^ Signaling chemicals such as neurotransmitters and neuropeptides are released into extracellular space through non‐synaptic vesicular fusion within axon varicosities (boutons). After being released into the intracellular fluid, the signaling molecules are transported to receptors on the targeted tissues via energy gradients, an intercellular communication process known as volume transmission.^[^ ^14^ ^]^ Peptidergic neurons are found in the periosteal cellular layer and sites of mineralization, contacting osteoblasts, and their precursors, osteoclasts, hematopoietic cells, as well as endothelial cells of intramedullary blood vessels.^[^ ^13^, ^14^, ^15^ ^]^ Sympathetic nerve fibers have been identified close to osteoblasts, osteoclasts and bone marrow adipocytes.^[^ ^13^, ^16^ ^]^ These nerve fibers can release neurotransmitters and neuropeptides that function locally on bone lineage cells and regulate bone metabolism.

#### 2.1.1 Norepinephrine

Norepinephrine (NE) is the most important neurotransmitter of the sympathetic nervous system within bone and exerts an overall inhibitory effect on bone remodeling. There are two classes of adrenergic receptors, *α*‐adrenergic receptors (*α*‐AR) and *β*‐adrenergic receptors (*β*‐AR).^[^ ^17^ ^]^

Presynaptic *α*‐ARs have the ability to control the release of NE.^[^ ^18^ ^]^ Other *α*‐ARs that are expressed in bone lineage cells such as osteoblast and osteoclast lineages affect the physiological activities of those cells through downstream signals once they are activated by NE.^[^ ^19^, ^20^, ^21^, ^22^, ^23^ ^]^ *α*‐AR signaling can upregulate proteins related to osteogenesis in MC3T3‐E1 osteoblastic cells and promotes bone formation.^[^ ^24^ ^]^ The intensity and duration of NE signals are regulated by feedback mechanisms through presynaptic *α*‐ARs, norepinephrine transporter,^[^ ^25^ ^]^ the cannabinoid system,^[^ ^26^, ^27^ ^]^ and neuropeptide Y.^[^ ^28^, ^29^ ^]^

*β*‐adrenergic receptors, which are expressed in osteoblasts and osteoclasts,^[^ ^30^, ^31^, ^32^, ^33^, ^34^, ^35^, ^36^ ^]^ are the principal adrenergic receptors involved in regulation of bone metabolism.^[^ ^30^, ^37^, ^38^, ^39^ ^]^ Norepinephrine can activate *β*‐ARs on osteoblasts to attenuate bone formation, and stimulate osteocytes to produce receptor activator of nuclear factor kappa‐Β ligand (RANKL); the latter in turn, can increase osteoclast differentiation.^[^ ^39^, ^40^ ^]^ Activation of *β*‐AR induces production of interleukin (IL)‐6 and IL‐11 in human osteoblasts. Both interleukins stimulate the differentiation of osteoclasts.^[^ ^41^ ^]^ *β*‐AR can directly regulate osteoclastogenesis through mediating generation of intracellular reactive oxygen species.^[^ ^42^ ^]^ *β*‐ARs are also involved in osteogenesis of mesenchymal stem cells, partly mediating via the cAMP/PKA signaling.^[^ ^43^ ^]^ Sometimes *β*1‐AR and *β*2‐AR signaling exert opposite effects on bone, with *β*1‐AR responsible for anabolic and *β*2‐AR responsible for catabolic activities.^[^ ^38^, ^44^, ^45^ ^]^

#### 2.1.2 Acetylcholine

The predominant neurotransmitter secreted by cholinergic nerves is acetylcholine (ACh). Once released, ACh exerts its cellular functions via nicotinic acetylcholine receptors (nAChRs), or muscarinic acetylcholine receptors (mAChRs).^[^ ^46^ ^]^ After release, ACh rapidly degrades into choline and acetate by acetylcholinesterase (AChE) and butyrylcholinesterase (BChE).^[^ ^46^ ^]^ Expression of these receptors by bone lineage cells are indicative of the regulatory roles of ACh on bone metabolism.^[^ ^47^, ^48^, ^49^, ^50^, ^51^, ^52^ ^]^

Through acting on the nAChRs or mAChRs expressed on bone lineage cells, ACh can have effect on the proliferation, differentiation of these cells as well as degree of ossification. Osteoclasts are the predominant type of bone lineage cells targeted by ACh through nAChRs expressed on their cell surfaces.^[^ ^53^, ^54^, ^55^ ^]^ Agonists of nAChRs have been shown to inhibit calcium oscillations in osteoclasts, and lacking *α*7 homomeric nAChR induced by knockout of *α*7 can reduce RANKL‐mediated in‐vitro osteoclastogenesis.^[^ ^54^ ^]^ Besides osteoclasts, in vitro data also demonstrated a direct action of cholinergic agonists on osteoblast proliferation and differentiation.^[^ ^55^ ^]^ In addition, the cholinergic nerve system may favor bone mass accrual directly or indirectly through a M3R‐dependent suppression of sympathetic signaling.^[^ ^56^, ^57^, ^58^ ^]^ Knockout of M3R led to declined trabecular bone volume, surface, and a higher trabecular pattern factor with increased number of osteoclasts.^[^ ^57^ ^]^ Skeletal embryonic development has been shown to depend heavily on cholinergic mechanisms, with beads soaked in ACh accelerating in ovo bone formation after implanted into chicken limb anlagen.^[^ ^59^, ^60^ ^]^

#### 2.1.3 Calcitonin Gene‐Related Peptide

Calcitonin gene‐related peptide (CGRP) is the major neuropeptide secreted by sensory nerves.^[^ ^61^, ^62^ ^]^ The neuropeptide modulates bone metabolism by stimulating osteoblast differentiation via the upregulation of transcription factor‑4 (ATF4) and osteocalcin, and inhibiting osteoprotegrin (OPG)/RANKL‑mediated osteoclastogenesis.^[^ ^63^ ^]^ Bone marrow mesenchymal stem cells (BMSCs) exposed to CGRP got improved proliferation, recruitment to ossification site and osteogenic differentiation, with enhanced expression of alkaline phosphatase and Runt‐related transcription factor 2 (Runx2).^[^ ^64^ ^]^ Receptors for CGRP are highly expressed on differentiating BMSCs; osteogenic differentiation of BMSCs is promoted by CGRP via the Wnt/*β*‐catenin signaling pathway.^[^ ^65^ ^]^ The neuropeptide also regulates communication between immune cells and osteoblasts to exert an anti‐resorptive effect on bone metabolism.^[^ ^66^ ^]^ Sensory denervation or the use of CGRP antagonists suppresses bone remodeling.^[^ ^67^ ^]^ Increase in CGRP concentration has been demonstrated after bone compression, which is suggestive of the role of sensory nerves in the adaptive response of bone to environmental mechanical stresses.^[^ ^68^ ^]^

#### 2.1.4 Substance P

Substance P (SP), secreted by sensory nerves, can enhance bone formation by increasing cAMP production, promoting osteoblast differentiation and enhancing BMP‐2 secretion.^[^ ^69^, ^70^ ^]^ However, some studies also found that the neurokinin‐1 receptor, which is expressed on osteoclasts, can stimulate bone resorption when activated by SP.^[^ ^71^ ^]^ This seemingly opposing effects of SP on bone remodeling may be attributed to its concentrations with 10^−8^ m as a watershed. Osteoblast differentiation and bone matrix mineralization are enhanced when SP concentration is higher than 10^−8^ m.^[^ ^71^, ^72^ ^]^ Conversely, osteogenic differentiation is blocked when SP concentrations is lower than 10^−8^ m.^[^ ^73^ ^]^ Exacerbated bone loss was identified in ovariectomy‐induced osteoporosis when SP signaling was blocked by its receptor antagonist, showing the significance of SP for maintaining normal bone mass.^[^ ^74^ ^]^ This conclusion coincides with impaired biomechanical and structural bone parameters which were observed in tachykinin 1‐deficient mice that lack SP.^[^ ^70^ ^]^

Crosstalk between SP and CGRP renders the role of neuropeptides on bone metabolism more complicated. Although both SP and CGRP enhance BMP2 signaling and mineralization in vitro, co‐stimulation of osteoblasts with SP and CGRP down‐regulates BMP2‐induced bone differentiation.^[^ ^75^ ^]^ The effect of SP plus CGRP contrary to the respective effects of SP or CGRP indicates possible interactions between these two neuropeptides, which lead bone remodeling from pro‐synthesis to pro‐resorption.

#### 2.1.5 Neuropeptide Y

Neuropeptide Y (NPY) is a neural signaling molecule that often accompanies NE release in sympathetic nerves. This neuropeptide signals through five receptors, among which Y1R and Y2R are often involved in bone remodeling.^[^ ^76^ ^]^ Neuropeptide Y acts both centrally and peripherally to influence bone metabolism, with central regulation of bone metabolism mostly via Y2R receptors and peripheral regulation mediated through Y1R receptors. Inhibition of Y1R receptor was shown to increase osteoblastic differentiation.^[^ ^76^ ^]^ Knockout or the use of antagonists on Y1R receptors resulted in higher bone mass with increased bone formation.^[^ ^77^, ^78^ ^]^ These studies demonstrate the negative regulatory roles of NPY on bone mass. Some studies also show that the neuropeptide exert sex‐specific effects on bone, with its function depending on certain sex hormones.^[^ ^79^ ^]^

#### 2.1.6 Vasoactive Intestinal Peptide

Vasoactive intestinal peptide (VIP) is often co‐released from cholinergic nerve terminals with ACh, acting through the VIP receptors VPAC_1_ and VPAC_2_.^[^ ^80^ ^]^ The neuropeptide can stimulate functional receptors on osteoblasts, promote cAMP production and downregulate osteoclast production.^[^ ^81^, ^82^ ^]^ Vasoactive intestinal peptide also promotes osteogenic differentiation of bone lineage cells, stimulates angiogenesis with enhanced tube formation of endothelial cells, and increases vascular endothelial growth factor expression in BMSCs.^[^ ^83^ ^]^

### 2.2 Axon Guidance Factors

Axon guidance factors are multifunctional proteins that play dynamic roles in the regulation of various physiological and pathological processes. These guidance cues are highly‐active during nerve growth or regeneration, which are likely to be another critical kind of components in the bone microenvironment, bridging peripheral nerves with the physiological and pathological processes manifested in bone metabolism.

#### 2.2.1 Sema3A and Sema4D

Expressed by both sensory and motor nerves, semaphorin 3A (Sema3A) is a regulator of axonal outgrowth, which induces collapse of the growth cone (cytoskeleton‐supported extension of a developing or regenerating neuron seeking its synaptic target) in sensory and sympathetic neurons.^[^ ^84^ ^]^ The receptor of Sema3A, neuropilin‐1, which binds to plexin‐A when taking function, has been demonstrated to be expressed by various bone lineage cells.^[^ ^85^, ^86^, ^87^, ^88^ ^]^ Bone mass was markedly lower in neuron‐specific Sema3A‐deficient mice, indicating that Sema3A secreted by peripheral nerves is involved in bone remodeling.^[^ ^89^ ^]^ The regulatory roles of Sema3A on bone formation can even offset the effects of sex hormones. Induction of site‐specific Sema3A overexpression ameliorated bone loss in osteoporotic ovariectomized mice, with suppression of bone resorption and increase in bone formation.^[^ ^90^ ^]^

Semaphorin 4D (Sema4D) and its receptors plexin‐B1 and plexin‐B2 are expressed in the embryonic dorsal root ganglion.^[^ ^91^ ^]^ Semaphorin 4D is also selectively expressed in oligodendrocytes and myelin of the central nervous system, which can induce growth cone collapse in those developing axons.^[^ ^92^ ^]^ Receptors of Sema4D are well expressed in bone lineage cells.^[^ ^87^, ^93^ ^]^ Studies found the upregulation of bone mass in Sema4D knockout animals compared to wild ones.^[^ ^94^, ^95^, ^96^ ^]^ Semaphorin 4D can inhibit expression of osteoblast differentiation markers and reduce the formation of mineralized nodules in vitro and in vivo.^[^ ^94^, ^95^ ^]^ The function of Sema4D on bone remodeling has been reported to be sex‐dependent.^[^ ^96^ ^]^ For post‐menopausal women, higher serum Sema4D was found among those with osteoporosis.^[^ ^97^ ^]^ Interestingly, increased risk of osteoarthritis has been demonstrated in humans with congenital defects derived Sema4D loss.^[^ ^98^ ^]^

#### 2.2.2 Netrin‐1

Netrin‐1 is expressed in Schwann cells with upregulation during peripheral nerve repair.^[^ ^99^ ^]^ The receptor of netrin‐1, Unc5B, is well expressed in bone lineage cells.^[^ ^100^, ^101^, ^102^, ^103^ ^]^ Netrin‐1 has been reported to stimulate bone formation via a mechanism involving osteoclast‐derived bone morphogenetic protein (BMP)‐2.^[^ ^100^, ^104^ ^]^ Some studies also found that Netrin‐1 regulates osteoclast differentiation, since Netrin‐1 binding to Unc5b can promote osteoclast differentiation both in vitro and in vivo through altering cytoskeletal assembly.^[^ ^101^ ^]^ In an osteocyte cell culture study that selectively patterned a cell‐growing scaffold with netrin‐1 via inkjet printing, elongation of osteocyte cell bodies or dendritic processes were identified in destined directions, offering solid evidence that axonal guidance cues serve as extrinsic factors for osteocyte dendrogenesis.^[^ ^105^ ^]^

#### 2.2.3 Slit‐3

Slit‐3 is expressed by the cell bodies and axons of sensory and motor neurons, satellite cells of the dorsal root ganglion, Schwann cells and fibroblasts of peripheral nerves.^[^ ^106^ ^]^ Roundabout axon guidance receptor 1 (ROBO1), the receptor of Slit‐3, is expressed on bone lineage cells, including osteoblasts and osteoclasts.^[^ ^107^, ^108^, ^109^ ^]^ Slit‐3 can stimulate osteoblast migration and proliferation by activating *β*‐catenin. It may also function in inhibiting bone resorption by suppressing osteoclast differentiation. Correlation was found between the circulating level of Slit‐3 and bone mass in postmenopausal women.^[^ ^110^ ^]^

### 2.3 Neurotrophins

Neurotrophins are factors capable of supporting neural activities, including axonal growth, synaptic plasticity, cell survival, differentiation, and myelination.^[^ ^111^, ^112^ ^]^ The neurotrophins family encompasses nerve growth factor (NGF), brain‐derived neurotrophic factor (BDNF), glial cell line‐derived neurotrophic factor (GDNF), neurotrophin 3 (NT‐3), and neurotrophin 4/5 (NT‐4/5).^[^ ^113^ ^]^ Apart from their functions in regulating neural system, the neurotrophins NGF and BDNF are also responsible for mediating bone metabolism, functioning as another kind of important connections between peripheral nerves and the skeleton.

#### 2.3.1 Nerve Growth Factor

Nerve growth factor is often upregulated during nerve regeneration.^[^ ^114^ ^]^ In addition to its neurotrophic capabilities, NGF influences bone remodeling through regulating osteogenesis and bone resorption. Tropomyosin receptor kinase A (TrkA) and the low‐affinity p75 neurotrophin receptor (p75NTR), the receptors for NGF, are expressed on osteoblasts, osteoclasts, and chondrocytes.^[^ ^115^, ^116^, ^117^, ^118^, ^119^, ^120^, ^121^ ^]^ In vitro studies showed that NGF prevents apoptosis of osteoblastic cells^[^ ^122^ ^]^ and induces differentiation of cultured osteoblastic cell lines.^[^ ^123^ ^]^ Enhanced osteoblast growth and differentiation was observed when bone repair scaffolds such as porous biphasic calcium phosphate was combined with NGF.^[^ ^124^ ^]^ Nerve growth factor also induces osteoclastogenesis in a RANKL‐independent manner.^[^ ^123^, ^125^ ^]^

#### 2.3.2 Brain‐Derived Neurotrophic Factor

This neurotrophic factor is localized to a subpopulation of dorsal root ganglion neurons from which the sensory nerves within skeleton are derived. Similar to NGF, BDNF regulates new bone formation through induction of osteoblast proliferation and differentiation.^[^ ^123^ ^]^ Brain‐derived neurotrophic factor also enhances RANKL secretion by human BMSCs, which contributes to osteoclastogenesis.^[^ ^126^ ^]^ In vivo experiment showed that BDNF promoted osteosclerosis after cortical osteotomy.^[^ ^127^ ^]^ TrkB, which binds exogenous BDNF, is expressed by chondrocytes, osteoblasts, osteoclasts, and cementoblasts. This finding is evocative of the potential regulatory role of BDNF on bone metabolism.^[^ ^116^, ^122^, ^128^, ^129^ ^]^ Incorporation of BDNF in bioactive scaffolds promoted healing of femoral metaphyseal fracture with augmented bone formation.^[^ ^130^ ^]^

### 2.4 Participation of Cells from Nerves

**Figure 2:**
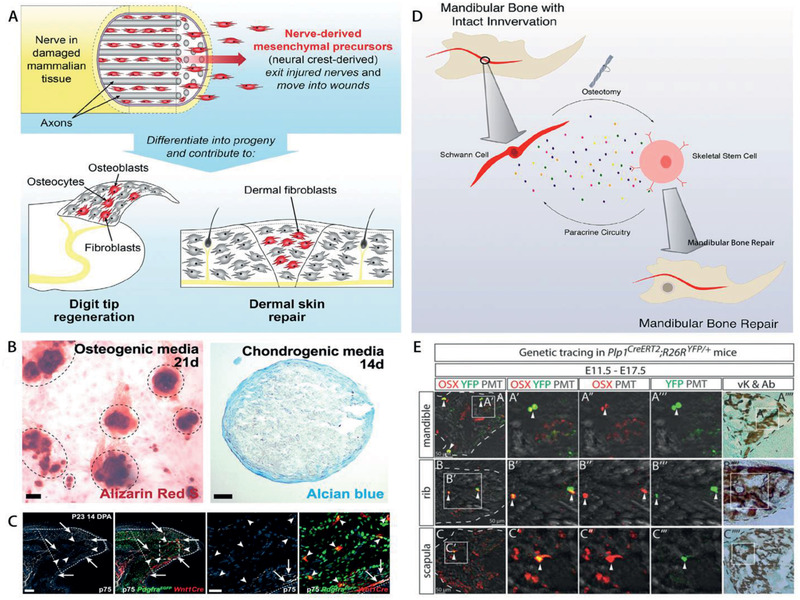
Participation of cells from nerves in embryonic bone development and bone regeneration. A) Nerve‐resident mesenchymal cells contribute to bone regeneration. B) Pdgfra‐positive mesenchymal cells can adopt features of bone and cartilage lineage cells when differentiated in vitro. C) Many TdT‐positive, Pdgfra‐EGFP‐positive, p75‐negative nerve‐derived mesenchymal cells are found located within and immediately adjacent to the regenerating bone. D) Mouse skeletal stem cells rely on paracrine factors secreted by Schwann cells as the underlying mechanism for mandibular bone regeneration. E) Schwann cell precursors generate osteoprogenitor cells and osteocytes in facial region and trunk during murine embryonic development. Schwann cell precursors progeny in Plp1CreERT2; R26RYFP/+ embryos traced from E11.5 to E17.5 were positive for osteoprogenitor marker OSX in the ossified parts of mandible, rib, and scapula. (A–C) Reproduced with permission.^[^ ^134^ ^]^ Copyright 2018, Elsevier. (D) Reproduced under the terms of a Creative Commons Attribution license (CC‐BY‐4.0).^[^ ^136^ ^]^ Copyright 2019, The Authors. Published by Elsevier. (E) Reproduced with permission.^[^ ^135^ ^]^ Copyright 2019, The Authors. Published by National Academy of Sciences.

Nerves have recently been shown to play a direct role in the bone healing process via contribution of nerve‐resident mesenchymal cells (**Figure** **2**A).^[^ ^131^ ^]^ Mesenchymal cells in nerves have long been thought to provide support and protection for afferent and efferent axons.^[^ ^132^, ^133^ ^]^ When nerves are damaged, Pdgfra‐positive mesenchymal cells from all three sheaths including endoneurium, perineurium, and epineurium were found to initiate proliferation.^[^ ^134^ ^]^ Interestingly, when differentiated in vitro, Pdgfra‐positive mesenchymal cells can adopt features of bone and cartilage lineage cells, showing their capability in participating bone repair (Figure 2B). And when nerve containing labeled mesenchymal cells gets transplanted into a mouse with bone scratch injury, Pdgfra‐positive mesenchymal cells were found to migrate into the damaged bone area and participate in healing.^[^ ^134^ ^]^ Pdgfra‐positive mesenchymal cells in the endoneurium also migrated out of the nerve in toe‐tip‐amputated mice, which contributed to the regeneration of the blastema and a significant proportion of cells within the regenerated bone (Figure 2C).^[^ ^134^ ^]^

Schwann cell precursors, a kind of immature, multipotent peripheral glial cells, can detach from nerve fibers and become mesenchymal cells, which then differentiate into chondrocytes and mature osteocytes to assist in embryonic skeletal development (Figure 2E).^[^ ^135^ ^]^ Paracrine factors released by Schwann cells including platelet‐derived growth factor‐AA (PDGF‐AA), parathyroid hormone (PTH), and oncostatin M (OSM) are critically important for proper stem cell function in bone healing, even supporting the healing of denervated mandibles (Figure 2D).^[^ ^136^ ^]^

## 3 Possible Signals from Bone to Nerves within Bone

Bone accommodates peripheral nerves, serves as a scaffold and provides cues in different forms to influence the physiological activities of peripheral nerves within bone.

### 3.1 Chemical Cues

Chemical cues, in the form of bioactive signaling factors that are secreted by bone lineage cells, can affect the physiological activity of nerves (Table 1). They function as mediators of the crosstalk between nerves and bone.

#### 3.1.1 Release of Axon Guidance Factors by Bone Lineage Cells

Axon development requires the concerted efforts of axon guidance factors for precise control of axonal outgrowth and targeted innervation. Expression of Sema3A has been identified in chondrocytes, osteoblasts, and osteoclasts.^[^ ^137^ ^]^ In mandibular neurotrophism, Sema3A expression is maintained in bone cells through sympathetic signal stimulation, which in return, balances sympathetic and sensory neuron infiltration.^[^ ^138^ ^]^ Sema3A secreted by bone lineage cells regulates neural activities through its receptor, neuropilin‐1.^[^ ^139^, ^140^, ^141^, ^142^, ^143^ ^]^ Semaphorin 4D is strongly expressed by osteoclasts but not osteoblasts.^[^ ^87^, ^95^, ^96^, ^144^ ^]^ Sema4D derived from skeletal tissues can also modulate neural activities through receptors expressed on nerves.^[^ ^145^, ^146^, ^147^ ^]^ Some studies opine that Slit‐3 is secreted by osteoclasts, with increased production during osteoclast differentiation.^[^ ^110^ ^]^ A recent study addressed the controversial proposition of the source of Slit‐3 within bone and revealed that osteoclasts do not produce Slit‐3 protein while osteoblasts are the true physiological sources.^[^ ^109^ ^]^ Slit‐3 axon guidance cues emitting from the skeleton are sensed by nerves through the receptors, Robo1.^[^ ^148^, ^149^, ^150^, ^151^, ^152^ ^]^ Ephrin B2, an Eph receptor‐interacting protein which navigates nerve growth, can be identified from osteoclasts and osteoblasts.^[^ ^153^ ^]^ Osteoblasts produce Netrin‐1, the receptor of which is well expressed in the nervous system.^[^ ^154^, ^155^, ^156^ ^]^ Netrin‐1 has also been detected in osteoclasts; knockout of Netrin‐1 in osteoclasts abrogates sensory innervation into porous endplates.^[^ ^157^ ^]^ Apart from Netrin‐1, osteoblasts produce other axon guidance factors such as Netrin‐4^[^ ^158^ ^]^ and Ephrin type‐B receptor 4 (EphB4).^[^ ^151^ ^]^ These axon guidance factors derived from bone lineage cells play the roles of regulating neural activities, acting as signals from skeleton to neural system. The same kind of bioactive molecules within the intrabony microenvironment may serve for the diversified roles, transporting the information between these two systems.

#### 3.1.2 Release of Neurotrophins by Bone Lineage Cells

**Figure 3:**
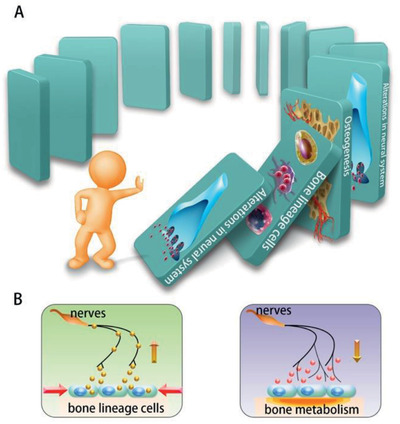
A) The communication between bone and intrabony nerves is like Dominoes. B) In response to stimulation from environment, bone lineage cells can release signals to function on nerves within bone. Consequently, the affected nerves may send out signals to regulate the bioactivities of the bone.

Neurotrophins secreted by the skeleton may function in controlling the amount and type of skeletal innervation. Through activating distinct tyrosine kinase receptors, neurotrophins can promote neuronal survival and growth. Nerve growth factor (NGF) plays an important role in the process of nerve outgrowth and innervation of target tissues, regulating nerve sprouting as well as neuropeptide expression and release through the receptors expressed in nervous system.^[^ ^118^, ^159^, ^160^, ^161^, ^162^, ^163^, ^164^ ^]^ The NGF‐TrkA signaling axis is critical for proper invasion of the neurovasculature.^[^ ^165^ ^]^ Nerve growth factor expression is upregulated during cell proliferation or upon outside loading.^[^ ^122^, ^166^, ^167^, ^168^ ^]^ During bone repair, NGF is also highly expressed in the fracture callus, indicating the correlation between the bone repair and nerve growth.^[^ ^169^ ^]^ Expression of NGF is enhanced in osteoblasts under loading stimulation; the NGF activated TrkA on periosteal sensory nerves and resulted in load‐induced nerve sprouting.^[^ ^167^ ^]^ The process of osteoblast‐sensory nerve‐osteogenesis demonstrates the complicated crosstalk between bone and nerves, with no distinct edges between bone affecting intrabony nerves and nerves affecting the bone. As is indicated in **Figure** **3**, the communication between bone and intrabony nerves is like Dominoes with interlocking consequences one after another once triggered by certain stimulation.

Brain‐derived neurotrophic factor and its receptor, TrkB, are also localized to fracture bone tissues during the early stage of bone repair. This neurotrophic factor concentrates predominantly in endothelial and osteoblastic cells in granulation tissue, at the margins of woven bone, while it is absent from chondrocytes and mature bone.^[^ ^121^, ^170^, ^171^ ^]^ The osteocyte‐like cell line OmGFP66 can express both NGF and BDNF.^[^ ^172^ ^]^ When released, BDNF attaches to receptors expressed in the nervous system to modulate its activities.^[^ ^163^, ^173^, ^174^, ^175^ ^]^ Taken together, neurotrophins provide mutual support for bone growth and nerve growth, especially during the process of hard tissue repair.

#### 3.1.3 Release of Inflammation‐Related Factors by Bone Lineage Cells

Sometimes, the participation of immune system is needed as an integrative interface between the two systems: the bone and the peripheral nerves. Inflammation‐related factors, including both pro‐inflammation factors and anti‐inflammation factors, may be released into intrabony extracellular microenvironment by bone lineage cells to function on peripheral nerves within bone. Bone cells contribute to immune‐mediated regulation in the intrabony microenvironment by producing factors such as interleukin‐7 (IL‐7), delta‐like 4 (DLL4), CXC‐ chemokine ligand 12 (CXCL12), granulocyte colony stimulating factor (G‐CSF) and prostaglandin E2 (PGE2), which can affect nerves within the microenvironment when get released.^[^ ^176^ ^]^

For instance, osteocytes, the terminally differentiated osteoblast lineage cells embedded in bone, can produce G‐CSF, which modulates neural activities such as pain processing and nociceptor activation.^[^ ^176^, ^177^ ^]^ Prostaglandin E2 (PGE2) is the most abundant prostaglandin in the body, which often participates the regulation of inflammation‐related reactions. Interestingly, it also serves as a neuromodulator which can sensitize peripheral sensory neurons and inhibits sympathetic release of NE.^[^ ^178^, ^179^ ^]^ In addition, PGE2 can protect motor neurons such as central facial neurons by inhibiting neuron apoptosis.^[^ ^180^ ^]^ This kind of neuromodulator can be expressed and secreted by osteoblast, which therefore participates in regulating sensory nerves via activating the PGE2 receptor 4 (EP4), and inhibits sympathetic activity through the central nervous system.^[^ ^181^ ^]^ The PGE2‐EP4 sensory nerve axis regulates both nerve activity and mesenchymal stem cell differentiation in adult murine bone marrow.^[^ ^182^ ^]^

#### 3.1.4 Regulation of Neurotransmitters and Neuropeptides by Bone Lineage Cells

**Figure 4:**
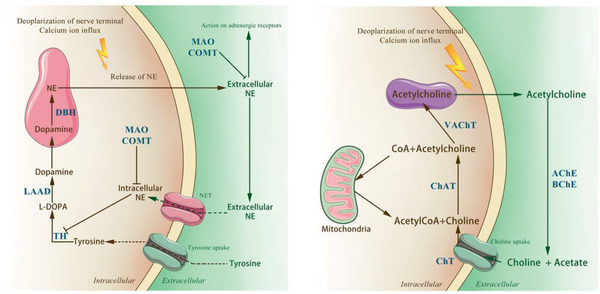
Metabolism of neurotransmitters. The images illustrate the synthesis, reuptake and catabolism of neurotransmitters. NE: norepinephrine. ACh: acetylcholine. TH: tyrosine hydroxylase. l‐DOPA: dihydroxyphenylalanine. DA: dopamine. LAAD: l‐aromatic amino acid decarboxylase. DBH: dopamine *β*‐hydroxylase. NET: NE transporter. MAO: monoamine oxidase. COMT: catechol‐*O*‐methyltransferase. ChAT: choline acetyltransferase. VAChT: vesicular ACh transporter. AChE: acetylcholine esterase. BChE: butyrylcholinesterases. CarAT: carnitine acetyl transferase.

Bone lineage cells have the ability to affect neurotransmitters in the bone microenvironment. For adrenergic nerves, bone lineage cells respond to AR agonists, with *α*‐AR and *β*‐AR expressed in osteoblasts, osteoclasts, chondrocytes and osteocytes.^[^ ^183^, ^184^ ^]^ According to metabolic process of NE (**Figure** **4**), these bone lineage cells lack the capability to generate catecholamines, with no evidence of expression of the enzyme dopamine veta‐hydroxylase, which catalyzes the conversion of dopamine to NE. Both catabolic enzymes monoamine oxidase (MAO)*α* and MAO*β* are expressed in osteoblast precursor cells and in fully‐differentiated osteoblasts, indicating that this cell lineage is able to catabolize NE.^[^ ^31^, ^46^ ^]^ Norepinephrine, which is regulated by bone in the microenvironment, may be sensed by receptors on nerves and plays potential regulatory roles on neural activities.^[^ ^185^, ^186^, ^187^, ^188^, ^189^, ^190^, ^191^, ^192^, ^193^ ^]^

Osteoblasts also express the cellular enzymes and transporters required to synthesize ACh.^[^ ^50^, ^51^ ^]^ Acetylcholine is degraded by AChE and BChE into choline and acetate (Figure 4). Both catabolic enzymes are expressed in osteoblasts.^[^ ^50^, ^51^ ^]^ The receptors of ACh are well located in nervous system, suggesting that skeleton may regulate neural activities through modulating the neurotransmitter ACh.^[^ ^194^, ^195^, ^196^, ^197^, ^198^ ^]^

Vasoactive intestinal peptide is expressed in isolated pure populations of osteoclasts.^[^ ^199^ ^]^ Expression of CGRP has been identified from primary human osteoblasts.^[^ ^200^ ^]^ Osteoblasts and osteocytes increase expression of SP and its receptor NK‐1 during exercise, linking mechanical responses to the osteogenic process.^[^ ^201^ ^]^ Neuropeptide Y is also produced by osteocytes and osteoblasts.^[^ ^202^ ^]^ The specific functions of the phenomenon remain unknown and need further investigation.

#### 3.1.5 Bone Morphogenetic Proteins Affecting Nerves

Bone morphogenetic proteins (BMPs) released from bone tissue and have multiple functions in promoting the development of cartilage and bone.^[^ ^203^ ^]^ They bind to transmembrane type I and type II serine/threonine kinase receptors and stimulate the response of intracellular Smad effector proteins. The BMPs are involved in regulating peripheral nerves. Receptors for BMP‐2 are expressed in nociceptive neurons of the dorsal root ganglion as well as in the pain processing lamina located at dorsal horn of the spinal cord.^[^ ^204^ ^]^ Potent neuroinflammatory responses were observed when rhBMP‐2 encapsulated absorbable collagen sponges were implanted into the spinous processes of adult rats.^[^ ^205^ ^]^ The BMPs such as BMP‐7 also play critical roles in regulating the development of sympathetic neurons through mediating early stages of dendritic growth in sympathetic neurons.^[^ ^206^, ^207^ ^]^ In addition, they have been reported to regulate the expression and release of neurotransmitters or neuropeptides as well as the differentiation of neural cells.^[^ ^208^, ^209^ ^]^

### 3.2 Mechanical Cues

Bone is a tissue that dynamically adapts to various stimulus such as mechanical loads that occur in daily life by remodeling.^[^ ^210^ ^]^ These alterations in mass and architecture are sensed by peripheral nerves within bone as mechanical cues. The mechanical cues, in turn, influence the activities of intrabony nerves.

Compression or traction forces exerted on the neurons by surrounding tissues contribute to the shaping of neuronal morphology and connectivity.^[^ ^211^ ^]^ Peripheral nerves within bone are constantly exposed to mechanical stresses associated with bone growth. Nerve elongation occurred along with bone regeneration during bifocal distraction osteogenesis.^[^ ^212^ ^]^ Peripheral nerve growth was promoted in a rabbit limb lengthening model, with compensatory increase of inter‐nodal length in axons in proportion to the mechanical strain.^[^ ^213^ ^]^ An in vitro study reported that cell stretch alone, in the absence of other stimulatory factors, induced significantly longer neurite growth.^[^ ^214^ ^]^ Other studies also reported enhanced neural stem cell differentiation and guided directionality of migrating neurons under mechanical tension.^[^ ^215^, ^216^ ^]^ Ultrasound stimulation, which exerts alternating compression and tension on nerves, can further promote axon regeneration compared with nerve conduit implantation alone.^[^ ^217^ ^]^

The stiffness of the peripheral environment can also be sensed by nerves and has an effect on nerve growth.^[^ ^218^, ^219^, ^220^ ^]^ Differentiation of mesenchymal stem cells into neuronal phenotype was promoted when cultured on deformable substrates instead of stiffer substrates.^[^ ^221^, ^222^ ^]^ The nerve guidance tube is a type of nerve repair scaffold that functions like peripheral tissues. The ideal elastic modulus of nerve guidance tubes has been reported to be 8–16 MPa.^[^ ^223^ ^]^

The microstructural features within the skeleton also affect nerves. Continuous formation and resorption of bone can cause changes in bone mass as well as wall thickness, porosity degree and pore size.^[^ ^224^ ^]^ The hole size of cortical bone ranges from 10 to 50 µm^[^ ^225^, ^226^ ^]^ and affects nerve growth. Trabecular bone possesses pore diameters ranging between 300 and 600 µm.^[^ ^226^, ^227^ ^]^ High porosity with high permeability that enables a sufficient supply of nutrients and oxygen is beneficial to nerve growth. The optimal structural parameters for peripheral tissues serving as nerve conduits are 80% porosity degree, 10–38 µm pore size and 0.6 mm wall thickness.^[^ ^228^ ^]^ Different areas within the skeleton with different porosity, pore size as well as topography may create highly variable effects on nerve growth.^[^ ^229^, ^230^ ^]^

### 3.3 Electrical Cues

Within the human body, endogenous electric fields regulate cellular behavior during tissue development or regeneration.^[^ ^231^, ^232^, ^233^, ^234^ ^]^ The piezoelectric and flexoelectric nature of bone also endow the hard tissue with additional electrical signals under mechanical stimulation,^[^ ^235^, ^236^ ^]^ which may affect physiological activity of nerves.^[^ ^237^ ^]^

Electrical signals not only serve as information that neurons deliver, but also function in regulating nerve growth as well as other neural physiological activities. Application of direct current electric fields has been reported to guide the migration of stem cell‐derived neural cells.^[^ ^238^ ^]^ Electrical stimulation may speed up axon regrowth,^[^ ^239^ ^]^ attenuate the damaged regeneration milieu, as well as improve sensory and motor nerve repair.^[^ ^240^, ^241^ ^]^ Nerve fiber density is increased under electrical stimulation, which, in turn, increases nerve functionality.^[^ ^242^ ^]^ Electrical stimulation of the limb also inhibited neuroma formation.^[^ ^243^ ^]^ Appropriate electrical stimulation improved nerve maturation and inhibited inflammatory reactions.^[^ ^244^, ^245^ ^]^ However, definite conclusion of the function of electrical signal from bone to intrabony nerves is still needed.

## 4 Crosstalk between Bone and Intrabony Nerves in Pathophysiological Conditions

Bilateral communication between bone and nerves occurs in different disorders. Some of these conditions are associated with both bone and peripheral neural abnormalities.

### 4.1 Osteoporosis

Osteoporosis is a group of distinct pathological conditions that features low bone mass and microarchitectural deterioration. These conditions often result in increased bone fragility and fracture susceptibility (see ref. ^[^ ^246^ ^]^ for review). Primary osteoporosis results from estrogen deficiency. This condition frequently occurs in post‐menopausal women, or is associated with aging in both sexes after the age of 60 (see ref. ^[^ ^247^ ^]^ for review). Secondary osteoporosis is caused by an underlying etiology such as Cushing's syndrome, or occurs after prolonged treatment with glucocorticoids (see ref. ^[^ ^248^ ^]^ for review).

**Figure 5:**
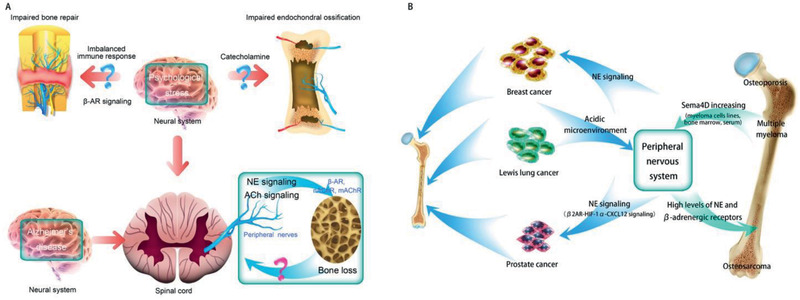
A) Possible crosstalk between bone and peripheral nerves in patients with psychological stress, Alzheimer's disease, osteoporosis, and impaired bone repair and endochondral ossification. B) Possible crosstalk between bone and peripheral nerves within the skeleton in patients with bone‐related tumor. NE: norepinephrine.

Psychological stress may be one of important medical conditions that contribute to reduced bone mineral density.^[^ ^249^ ^]^ While psychological stress and osteoporosis are different diseases which occur via distinct mechanisms, there are several factors that overlap between psychological stress and osteoporosis including glucocorticoids, catecholamines, insulin‐like growth factors, and so on.^[^ ^250^ ^]^ Psychological stress can accelerate bone loss and osteoporotic fractures through immune and endocrine mechanisms.^[^ ^251^ ^]^ The relationship between psychological stress and osteoporosis has been demonstrated with chronic mild stress, an established rodent model which leads to depression, causing reduction in osteoblast number, bone loss and reduced bone formation.^[^ ^252^, ^253^ ^]^ Peripheral nerves participate in regulating bone metabolism in individuals with psychological stress (**Figure** **5**A). One way in which psychological stress may impact osteoporotic disease risk and severity is through catecholamine‐induced activation of *β*‐adrenergic receptors on osteoblasts and osteoclasts, which can increase RANKL expression and result in osteoclast differentiation.^[^ ^250^ ^]^

Alzheimer's disease, an age‐related neurodegenerative disease notorious for destroying memory and cognition, is associated with a greater incidence of bone loss and skeletal fracture.^[^ ^254^, ^255^ ^]^ An abnormal central serotonergic regulating pathway has been identified in this disease. This pathway produces an overwhelming sympathetic nervous signaling tone, which activates *β*‐AR on the bone cells and enhance bone resorption.^[^ ^256^, ^257^, ^258^ ^]^ Cholinergic signals may also be related with osteoporosis. Significant decrease in mAChR M5 and mAChR M3 had been identified from osteoblasts in osteoporotic rats. These observations provid evidence for the involvement of AChR signaling in osteoporosis.^[^ ^194^, ^259^ ^]^

### 4.2 Osteoarthritis

**Figure 6:**
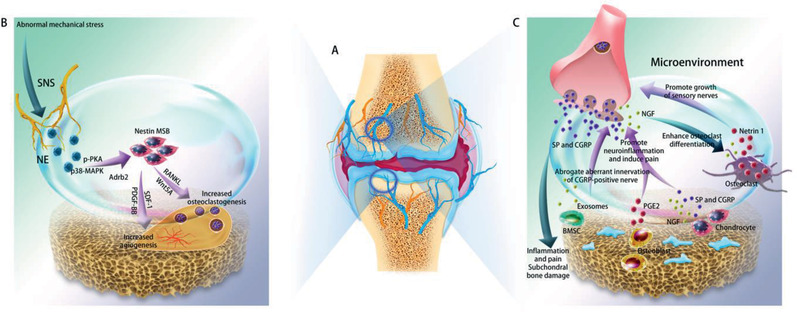
Crosstalk between bone and peripheral nerves within the skeleton during osteoarthritis. A) Innervation of cartilage, subchondral bone, synovium and the joint capsule during osteoarthritis. Blue indicates sensory nerves. Yellow indicates sympathetic nerves. B) Regulatory roles of sympathetic nerves on bone metabolism during osteoarthritis. NE released by sympathetic nerves play a regulatory role on bone metabolism during TMJ osteoarthritis caused by unilateral anterior crossbite through acting on *β*2AR (Adrb2). And selective deletion of *β*2AR (Adrb2) in nestin^+^ MSCs could attenuate progression of condylar subchondral bone loss as well as cartilage degradation. C: Bilateral communication between bone and peripheral sensory nerves during OA. SNS: sympathetic nerve system. NE: norepinephrine. Adrb2: *β*2‐adrenergic receptors. NGF: nerve growth factor. SP: substance P. CGRP: calcitonin gene‐related peptide. BMSC: bone marrow derived stroma cell.

Although osteoarthritis is a disorder of the articular cartilage, the condition may be considered an organ failure that involves abnormalities in the cartilage, bone, ligaments, synovium, and the joint capsule.^[^ ^260^ ^]^ Cartilage is neither vascularized nor innervated in healthy condition. However, sensory and sympathetic nerves have been identified in the articular cartilage in human tibiofemoral osteoarthritis (**Figure** **6**A).^[^ ^261^ ^]^ Increase in sensory nerve innervation was identified in the subchondral zone of a joint during osteoarthritis, extending into the calcified cartilage zone or close to the articular cartilage (Figure 6A). The concentration of sensory neuropeptides increases in the synovial fluid when osteoarthritis becomes more severe.^[^ ^262^, ^263^, ^264^ ^]^ The concomitant changes in peripheral nerves with joint pathology are indicative of the link between bone and nerves in osteoarthritis.

Peripheral nerves serve as the regulator of joint disorder during this pathophysiological process (Figure 6B,C). Sympathetic signaling is related to subchondral bone loss during osteoarthritis of the temporomandibular joint (TMJ) (Figure 6B).^[^ ^265^, ^266^, ^267^ ^]^ During osteoarthritis, cartilage metabolism is also modulated by neuropeptides released by sensory nerves including SP and CGRP.^[^ ^268^ ^]^ Osteoarthritis is accompanied by up‐regulation of CGRP, TrkA, as well as enlargement of the soma size of neurons expressing CGRP in both the joint and subchondral bone.^[^ ^269^ ^]^ Blocking CGRP resulted in inhibition of subchondral bone sclerosis during the progression of osteoarthritis.^[^ ^270^ ^]^ Increased secretion of CGRP and SP is closely related with inflammation and pain during osteoarthritis.^[^ ^271^, ^272^ ^]^ In addition, NGF from sensory nerves participates in the regulation of osteoarthritis. The two receptors of NGF, TrkA and p75NTR, are expressed by joint chondrocytes.^[^ ^273^, ^274^ ^]^ Intra‐peritoneal injection of anti‐NGF antibody resulted in suppressed up‐regulation of CGRP in the dorsal root ganglion neurons.^[^ ^275^ ^]^ Application of anti‐NGF antibodies reduced the number of tartrate‐resistant acid phosphatase‐positive osteoclasts in the subchondral bone in a murine model of osteoarthritis.^[^ ^276^ ^]^

Skeletal tissues also affect peripheral nerves in osteoarthritis (Figure 6C). Osteoclasts secrete netrin‐1 to induce the growth of sensory nerves in the subchondral bone, through its “Deleted in Colorectal Cancer” receptor, during aberrant subchondral bone remodeling in osteoarthritis.^[^ ^277^ ^]^ Nerve growth factor is expressed by chondrocytes^[^ ^261^, ^262^ ^]^ and is increased in osteoarthritic cartilage.^[^ ^278^, ^279^ ^]^ Synthesis of NGF was found to be highly‐correlated with the extent of osteoarthritic cartilage degradation in humans.^[^ ^279^ ^]^ Increased mechanical loading stimulated expression and release of NGF by chondrocytes in patients with osteoarthritis.^[^ ^280^ ^]^ During osteoarthritis, osteoblasts are transformed into a low secretory subtype that produce high quantities of PGE2. These altered osteoblasts act as mediators that cause inflammation, alter innervation and promote subchondral bone sclerosis.^[^ ^281^ ^]^ The released PGE2 sensitizes dorsal root ganglia neurons through regulation of voltage‐gated sodium channel Na_V_1.8, which induces pain as well as expedites disease progression.^[^ ^282^ ^]^ During osteoarthritis, BMSCs release exosomes to abolish aberrant innervation of CGRP‐positive nerves and abnormal formation of H‐type vessels in the subchondral bone.^[^ ^283^ ^]^ Endogenously‐produced SP was also found in newborn murine costal and adult human articular chondrocytes.^[^ ^284^ ^]^ Chondrocytes isolated from human osteoarthritic articular cartilage produce SP and CGRP, which affect nerves in the microenvironment, and function together with the neuropeptides to regulate the joint disorder.^[^ ^284^ ^]^

### 4.3 Heterotopic Ossification

Heterotopic ossification (HO) refers to ectopic formation of bone in the extraskeletal tissues. These ossified structures severely incapacitate people in their daily life. In HO, stem cells and progenitors are recruited to the extraskeletal tissues and are exposed to osteoinductive factors. These cells undergo all stages of endochondral bone formation as well as ectopic ossification. The newly‐formed HOs are similar to skeletal bone, possessing a bone marrow cavity which can fuse with the normal skeleton. Within the general population, the risk of HO is approximately 5%, while 60% of military casualties are found with HO.^[^ ^285^ ^]^ Military traumatic injuries are often the direct result of blast or burn injuries, which may have dramatic effects on the peripheral and central nervous systems. This suggests a link between nerve stimulation and the high incidence of HO.^[^ ^285^ ^]^

**Figure 7:**
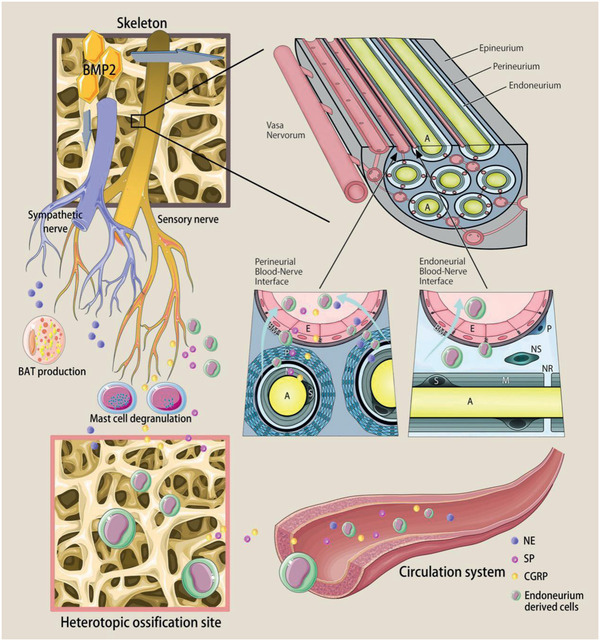
Crosstalk between bone and peripheral nerves within the skeleton during heterotrophic ossification (HO). Neuropeptides and neurotransmitters from peripheral nerves regulate BAT production and mast cell degranulation. Cells derived from peripheral nerves are transported to the HO site and function as osteoprogenitors. BMP2 from the bone matrix triggers the release of neuropeptides by peripheral nerves as well as increases the permeability of blood‐nerve barrier for cells and other biological factors. NE: norepinephrine. SP: substance P. CGRP: calcitonin gene‐related peptide. BAT: brown adipose tissue. BMP2: bone morphogenetic protein 2. [Some drawing elements are adopted from SMART Servier Medical ART under the terms of the CC‐BY Creative Commons Attribution 3.0 Unported license. (http://creativecommons.org/licenses/by/3.0/).] Adapted with permission,^[^ ^299^ ^]^ copyright 2018, Frontiers Media S.A.

Peripheral nerves play important roles in regulating HO (**Figure** **7**). First, peripheral nerves provide the cell source for HO. A significant number of cells within the endoneurium express the osteoblast‐specific transcription factor osterix, as well as neural crest markers such as platelet‐derived growth factor‐beta.^[^ ^286^, ^287^ ^]^ This growth factor is also a marker of human osteoprogenitors derived from traumatic neurogenic HO.^[^ ^288^ ^]^ These data suggest migration and expansion of cells derived from neurons into the ossification site. During embryonic development, neural crest cells transform into mesenchymal cells for osteogenesis. This process appears to be recapitulated during HO.^[^ ^289^, ^290^ ^]^ Expression of osteoblast‐specific transcription factors was identified in cells derived from the neural endoneurium after HO induction. Those transcription factors reappeared at the site of new bone formation after transient expression in the circulation.^[^ ^286^ ^]^ Most of the osteogenic precursors in neurogenic HO are endoneural progenitors. These multipotent cells differentiate into osteoblasts, chondrocytes, and brown adipocytes.^[^ ^291^ ^]^

Second, neuropeptides such as CGRP and SP released by sensory nerves are responsible for neuroinflammation. These neuropeptides recruit mast cells and stimulate the degranulation of mast cells to promote HO.^[^ ^287^, ^292^, ^293^ ^]^ Transient receptor potential cation channel V1 (TRPV1) sensory nerves play important roles in HO by secreting SP and CGRP. Mice lacking TRPV1 sensory nerves developed significantly less HO.^[^ ^287^, ^293^ ^]^ Sensory nerve terminals can further release SP and CGRP when activated by mast cell mediators, thereby creating a positive feedback loop for neurogenic inflammation.^[^ ^292^ ^]^

Third, the production of brown adipose tissue, which is essential for HO, is regulated by sympathetic nerves. Norepinephrine released by sympathetic nerves stimulates *β*3‐adrenergic receptors expressed on brown adipose cells to regulate proteins related to brown adipose tissue phenotype.^[^ ^294^ ^]^ Administration of *β*3‐adrenergic receptor agonists increased brown adipose tissue in mice, dogs, and primates.^[^ ^295^, ^296^ ^]^

Skeletal tissues also have reverse effects on peripheral nerves during HO (Figure 7). Traumatic injury causes release of BMP from the skeletal bone matrix.^[^ ^297^, ^298^ ^]^ Permeabilization of nerves after injury permits entry of BMP2. The BMP2 acts on the blood‐nerve barrier of peripheral nerves and causes nerve‐derived osteoprogenitors to exit the nerve and migrate to the site of ossification.^[^ ^299^ ^]^ The blood‐nerve barrier protects the endoneurium, which contains axons, Schwann cells, and neural crest‐derived progenitors, from the external environment but permits gas exchange and small molecules to permeate.^[^ ^299^, ^300^, ^301^, ^302^ ^]^ Recent studies showed that cells within the endoneurium of peripheral nerves undergo BMP‐Smad signaling, indicating that BMP may bind and activate its receptor.^[^ ^301^, ^303^ ^]^ The dose of BMP2 and the number of cells positive for BMP‐Smad signaling within the endoneurium were found to be correlated with the extent of HO.^[^ ^41^ ^]^ After activation by BMP2, a cascade of events occurs within the peripheral nerves to cause neuroinflammation, with elevated the level of SP and CGRP released by sensory nerves.^[^ ^304^ ^]^ Neuroinflammation is necessary for opening the blood‐nerve barrier.^[^ ^305^ ^]^ Nerve‐derived osteoprogenitors express Claudin‐5 when they cross the blood‐nerve barrier to the ossification site. Matrix metalloproteinase‐9 is highly expressed at the site where HO occurs; the proteinase is activated after opening of the blood‐nerve barrier.^[^ ^297^, ^306^, ^307^ ^]^ These data suggest that the crosstalk between peripheral nerves and bone tissue in HO may be a potential therapeutic target for HO intervention.

### 4.4 Psychological Stress‐Related Bone Abnormalities

Depression is responsible for abnormal emotional and physical symptoms that include loss of interest and dysregulated sleep. Anxiety may be accompanied by physical symptoms such as increased heart rate and breathlessness. These mood disorders often have a common cause, psychological stress.^[^ ^308^ ^]^ Apart from osteoporosis, psychological stress‐related bone abnormalities are also regulated by peripheral nerves (Figure 5A).

Induction of chronic psychological stress in adolescent mice reduced endochondral ossification in the growth plates, as well as reduced tibia and femur lengths.^[^ ^309^ ^]^ Another study examined the role of chronic psychosocial stress on bone repair.^[^ ^310^ ^]^ Chronic psychosocial stress also caused imbalanced immune response via *β*‐AR signaling. An augmented number of neutrophils were present in the early fracture hematoma. The latter was accompanied by disturbed fracture healing.^[^ ^310^ ^]^

### 4.5 Bone Related Tumors

Bone is one of the most common sites for cancer metastasis, in which many types of metastasized cancer have been found to be associated with peripheral nerves (Figure 5B). Stimulating *β*2AR in osteoblasts by neurotransmitters can increase bone vascular density and enhance the colonization of metastatic breast cancer cells.^[^ ^311^ ^]^ Norepinephrine secreted by sympathetic nerves can reactivate dormant, disseminated prostrate tumor cells in the bone marrow niche, directly stimulating the proliferation of prostate cancer cell via *β*2‐AR.^[^ ^312^ ^]^ Norepinephrine can also downregulate the secretion of the dormancy‐inducing molecule, growth arrest specific‐6 by osteoblasts, which then affect prostate cancer cell proliferation.^[^ ^312^ ^]^ Another study also reported augmented *β*2AR‐hypoxia‐inducible factor 1*α*‐CXC motif chemokine‐12 (CXCL12) signaling in osteoblasts, and the increased signaling facilitated migration, invasion and epithelial‐mesenchymal transition of prostate cancer cells.^[^ ^313^ ^]^ Therefore, whether affecting cancer cells directly or indirectly through mediation of osteoblasts, neurotransmitters are of unignorable roles to regulate intrabony microenvironment under the situation of metastasized tumors. The alterations within bone have reverse effects on intrabony peripheral nerves in occasions of metastasized tumors. For example, the intrabony acidic cancer microenvironment of Lewis lung cancer in bone metastasis can activate sensory nerves and augmented sensory nerve excitation, contributing to increased bone pain.^[^ ^314^ ^]^

Some bone tumors, such as multiple myeloma (MM) and osteosarcoma, are also suspected to be related to alterations in the peripheral nervous system, though the direct connection is still implicit and under investigation (Figure 5B). Increased expressions of Sema4D and its receptor Plexin‐B1 have been evaluated in myeloma cell lines in vitro as well as in the bone marrow plasma and serum of patients diagnosed with symptomatic MM.^[^ ^315^ ^]^ The expressions are also correlated with adverse myeloma features and enhanced bone resorption.^[^ ^315^ ^]^ Catecholamines and their receptors are regarded as potential molecular markers of osteosarcoma progression. Significantly higher levels of NE were found in the cancer sample compared to non‐oncological bone.^[^ ^316^ ^]^ Gene expressions of *β*‐adrenergic receptors were significantly higher in the tumorous tissue.^[^ ^316^ ^]^ These results suggest a potential link between peripheral nerves and bone tumors that requires further investigation.

### 4.6 Mutual Impact between Bone Repair and Nerve Growth

The crosstalk may exist between bone repair and nerve growth. As mouse toe tips do not regenerate properly in the absence of local nerve innervation, nerves play a significant role during tissue regeneration and nerves within bone are supposed to function as a mediator for bone regeneration.^[^ ^317^, ^318^ ^]^ After bone fracture, abundant sprouting of nerve fibers were seen in the callus, hyperplastic periosteum, and edge of fibrocartilage, and nerve fibers were sprouting into the fibrocartilage and new woven bone, prior to vascularization at early time points.^[^ ^319^, ^320^ ^]^ Sensory denervation as well as antagonism of CGRP can bring about suppression of bone reshaping and proinflammatory milieu evidenced by bone histomorphometry and immunohistochemistry during bone remodeling.^[^ ^67^ ^]^ A study investigated the effects of inferior alveolar nerve on new bone formation in rabbit and found that the loss of the sensory nerves could result in decreased new bone quality during the mandibular distraction osteogenesis.^[^ ^321^ ^]^ These phenomena indicate the participation of peripheral nerves during bone regeneration.

And intrabony microenvironment also exerts effects on nerve repair. Bone marrow stromal cells (BMSCs) play an important role in supporting nerve repair. After damage of the peripheral nervous system, complex and orchestrated cellular and molecular events occur to support nerve regeneration.^[^ ^322^ ^]^ When induced by Schwann cell‐derived exosomes, BMSCs differentiate into Schwann cells.^[^ ^323^ ^]^ Application of BMSC‐implanted scaffold with granulocyte colony‐stimulating factor has been shown to ameliorate the niche of neurotization and advance nerve regeneration substantially.^[^ ^324^ ^]^ The usage of low‐frequency pulsed electromagnetic field to simulate bone marrow‐derived mesenchymal stem cells promoted regeneration of crush‐injured rat mental nerve.^[^ ^325^ ^]^ In addition, BMSCs has been used to repair brachial plexus injury in rabbits, with enhanced nerve repair and improved nerve physiological functions.^[^ ^326^ ^]^

## 5 Triggering the Crosstalk through Implanted Scaffolds during Bone Regeneration

Some tissue engineering scaffolds possess the capacity to trigger and reproduce the natural communication between peripheral nerves and bone during bone repair. For example, a magnesium‐containing intramedullary nail was fabricated with the capacity to enhance femur fracture repair in rats with osteoporosis.^[^ ^327^ ^]^ Extracellular magnesium was found to enter sensory nerves within periosteum and activate secretion of CGRP by sensory nerves, which in turn enhanced osteogenic differentiation of periosteum‐derived stem cells and osteoblasts to promote bone repair.^[^ ^327^ ^]^ This scaffold takes the advantage of positive regulatory roles played by sensory nerves on bone formation, enhancing bone repair in an indirect manner.

In another study, a collagen bone substitute encapsulated with NGF was created with ability to stimulate neurogenesis and enhance the expression of pro‐angiogenesis growth factors. Enhanced bone formation was observed compared to the group without NGF encapsulation, providing the proof of indirect stimulation of bone repair through promoting neurogenesis.^[^ ^328^ ^]^ Scaffolds directly aim at repairing intrabony nerves can result in enhanced bone regeneration indirectly, compared to those using same materials without neurotrophic factors. Another biomimetic bone‐like system realized replication of the key hallmarks of the bone cellular and extracellular microenvironment through applying biomineralization and embedding osteoprogenitor, vascular, and neural cells within, which demonstrated harmonious osteogenesis, angiogenesis, and neurogenesis.^[^ ^329^ ^]^ However, further realization of simultaneous regeneration of bone and nerves need to first solve the different requirements of two or more tissues on internal microenvironment within scaffolds.

Inspirations acquired from nature's intriguing design of bilateral communication between peripheral nerves and the skeleton provide the backdrop for the design of better artificial bone implants for fracture repair. In the future, signals provided by implants may activate the peripheral nervous system to regulate bone regeneration, and may enhance the reinnervation of bone defects to improve long‐term prognosis. However, this idea is sill in its infancy. Further investigations of these novel conceptions are called upon in order to harness the benefits of mutual regulation of peripheral nerves and bone for the purpose of bone and nerve regeneration.

## 6 Concluding Remarks and Future Perspectives

The crosstalk between bone and nerves is discussed in the present review, with support of evidence derived from physiological and pathological events. Environmental cues in the form of chemical, mechanical, or electrical stimulation are precisely orchestrated to coordinate the metabolism and activities of bone and nerves. Occasionally, the direction of certain signals (i.e., from nerves to regulate bone or from bone to affect nerves) appears to be distinct, with clear derivation and targeting destinations in certain conditions. More commonly, however, the direction is not so clear cut to determine the direvation as well as the acting objects of signals within intrabony microenvironment. With only fragments of the complete story unveiled, downstream biological activities are still vastly unknown for many exchanged signals between bone and nerves. In the grand scheme of things, communication between bone and nerves is analogous to a subtle conversation that encodes complicated signal exchanges between the two entities.

Pharmaceutical approaches using agonists or antagonists, or the usage of signaling molecule knockout, are commonly employed in this field of study to investigate the roles played by chemical cues emitted from peripheral nerves or the skeleton. However, the overall effects of the agonist/antagonist on the physiological activities of aimed tissue, besides upregulating or downregulating targeted signaling, is sometimes far more complicated than a molecular biologist assumes or can comprehend, often resulting in the generation of ambiguous outcomes. Likewise, even with the utmost consideration in the design of a genetic alteration scheme, unpredictable changes may occur in peripheral nerves, skeleton or any other tissue during embryonic development, generating results that are either erroneous at best or completely misleading. In addition, delivery of a drug needs to simulate the natural growth or regeneration process. Accordingly, a drug‐encapsulated scaffold with controlled release of the designated drug is a more viable alternative. Besides, the implanted scaffold should not be merely aimed at tissue repair but should encompass elements that enable the intervention of pre‐determined experimental parameter for understanding of the consequent outcome. The use of scaffolds or/and implanted cells for creating a 3D environment may serve as an in vitro model for the study of pathophysiological process. At present, the investigations on pathophysiological process mainly depend on in vivo and in vitro studies, which refer to the animal models or the cell researchers respectively. However, organoids composed of cells and scaffolds may be a new road, which can mimic certain tissues or organs including epithelium and human brain, reproducing the procedure of disease progression in a biomimetic environment.^[^ ^330^, ^331^ ^]^ In vitro organoid models have been successfully applied to recapitulate the pathophysiological process of drug‐induced liver fibrosis, muscular dystrophies, tumor hypoxia and tumor‐immune interactions, and so on.^[^ ^332^, ^333^, ^334^ ^]^ In addition, it is exciting to see a novel callus‐mimicking organoid which consists of cells derived from human periosteum assembling microspheroids, capable of spontaneously bio‐assembling in vitro into large tissues to repair murine critical‐sized long bone defects.^[^ ^335^ ^]^ Since both bone (e.g., callus organoid) and nerves (e.g., brain organoid) can be bio‐mimicked in vitro, a more delicate 3D system containing both bone and nerves is hopeful to be created in the future to investigate the pathophysiological process of diseases related to bone and neural system as well as the mutual communication of bone and nerves. It is anticipated that unidentified dialogs between peripheral nerves and bone will be unveiled through the adoption of novel approaches that combine materials science with pathophysiological research.

**Figure 8:**
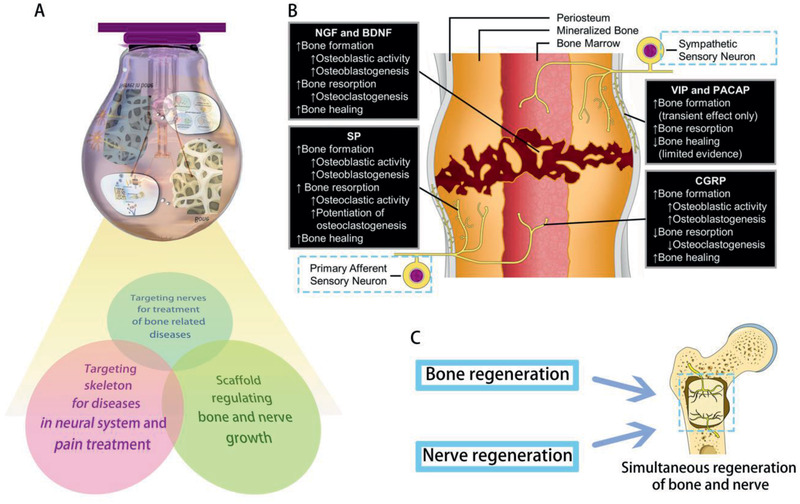
Future perspectives on targeting the crosstalk between bone and peripheral nerves for disease treatment or tissue regeneration. A) Schematic showing the future perspectives. B) Targeting peripheral nerves within bone may promote bone fracture healing. Blue dotted frames indicate the potential target. C) Schematic showing simultaneous regeneration of bone and nerves using implanted scaffolds. (B) Reproduced with permission.^[^ ^336^ ^]^ Copyright 2020, Elsevier.

Although reaping the benefits out of the sophisticated crosstalk between bone and nerves for treatment of diseases or tissue regeneration is highly‐esteemed, such a noble notion is still beyond reach by even the most understanding molecular biologist or genetic engineer. Nonetheless, conceptual perspectives do exist but require diligent investigations (**Figure** **8**). For bone‐related diseases such as osteoporosis, osteoarthritis, heterotopic ossification and bone tumors, the crosstalk between peripheral nerves and bone may be potential targets to regulate bone metabolism in a positive direction, as well as to alleviate pain in these pathophysiological conditions. It has been shown that targeting of specific signals emitted by peripheral nerves expedites healing of bone fracture.^[^ ^336^ ^]^ Altering the microenvironment of nerves within the skeleton may generate signals for the rehabilitation of degenerative nerve diseases that physicians are currently incapable of handling. Of note, some diseases of the central nervous system are associated with bone metabolism. For example, bone osteocalcin has been reported to modulate cognition and anxiety‐like behaviors.^[^ ^337^, ^338^ ^]^ Bone has been traditionally perceived as the supportive and protective organ. However, accumulating evidence indicates that bone, as an endocrine organ, may also be an invisible regulator of other body systems. Because of the crosstalk between bone and nerves, future design of bone repair materials should give priority to the development of materials that regenerate nerves as well as bone.

## Conflict of Interest

The authors declare no conflict of interest.
